# Detecting Artificial Intelligence–Generated Versus Human-Written Medical Student Essays: Semirandomized Controlled Study

**DOI:** 10.2196/62779

**Published:** 2025-03-03

**Authors:** Berin Doru, Christoph Maier, Johanna Sophie Busse, Thomas Lücke, Judith Schönhoff, Elena Enax- Krumova, Steffen Hessler, Maria Berger, Marianne Tokic

**Affiliations:** 1 University Hospital of Paediatrics and Adolescent Medicine, St. Josef-Hospital Ruhr University Bochum Bochum Germany; 2 Departement of German Philology, General and Comparative Literary Studies Ruhr University Bochum Bochum Germany; 3 Department of Neurology, BG University Hospital Bergmannsheil gGmbH Bochum Ruhr University Bochum Bochum Germany; 4 German Department, German Linguistics Ruhr University Bochum Bochum Germany; 5 German Department, Digital Forensic Linguistics Ruhr University Bochum Bochum Germany; 6 Department for Medical Informatics, Biometry and Epidemiology Ruhr University Bochum Bochum Germany

**Keywords:** artificial intelligence, ChatGPT, large language models, textual analysis, writing style, AI, chatbot, LLMs, detection, authorship, medical student, textual analysis, linguistic quality, decision-making, logical coherence

## Abstract

**Background:**

Large language models, exemplified by ChatGPT, have reached a level of sophistication that makes distinguishing between human- and artificial intelligence (AI)–generated texts increasingly challenging. This has raised concerns in academia, particularly in medicine, where the accuracy and authenticity of written work are paramount.

**Objective:**

This semirandomized controlled study aims to examine the ability of 2 blinded expert groups with different levels of content familiarity—medical professionals and humanities scholars with expertise in textual analysis—to distinguish between longer scientific texts in German written by medical students and those generated by ChatGPT. Additionally, the study sought to analyze the reasoning behind their identification choices, particularly the role of content familiarity and linguistic features.

**Methods:**

Between May and August 2023, a total of 35 experts (medical: n=22; humanities: n=13) were each presented with 2 pairs of texts on different medical topics. Each pair had similar content and structure: 1 text was written by a medical student, and the other was generated by ChatGPT (version 3.5, March 2023). Experts were asked to identify the AI-generated text and justify their choice. These justifications were analyzed through a multistage, interdisciplinary qualitative analysis to identify relevant textual features. Before unblinding, experts rated each text on 6 characteristics: linguistic fluency and spelling/grammatical accuracy, scientific quality, logical coherence, expression of knowledge limitations, formulation of future research questions, and citation quality. Univariate tests and multivariate logistic regression analyses were used to examine associations between participants’ characteristics, their stated reasons for author identification, and the likelihood of correctly determining a text’s authorship.

**Results:**

Overall, in 48 out of 69 (70%) decision rounds, participants accurately identified the AI-generated texts, with minimal difference between groups (medical: 31/43, 72%; humanities: 17/26, 65%; odds ratio [OR] 1.37, 95% CI 0.5-3.9). While content errors had little impact on identification accuracy, stylistic features—particularly redundancy (OR 6.90, 95% CI 1.01-47.1), repetition (OR 8.05, 95% CI 1.25-51.7), and thread/coherence (OR 6.62, 95% CI 1.25-35.2)—played a crucial role in participants’ decisions to identify a text as AI-generated.

**Conclusions:**

The findings suggest that both medical and humanities experts were able to identify ChatGPT-generated texts in medical contexts, with their decisions largely based on linguistic attributes. The accuracy of identification appears to be independent of experts’ familiarity with the text content. As the decision-making process primarily relies on linguistic attributes—such as stylistic features and text coherence—further quasi-experimental studies using texts from other academic disciplines should be conducted to determine whether instructions based on these features can enhance lecturers’ ability to distinguish between student-authored and AI-generated work.

## Introduction

The rapid development of artificial intelligence (AI) and the emergence of large language models (LLMs), such as ChatGPT, have increasingly blurred the lines between human-written and AI-generated text. This has created a significant challenge in identifying the authorship of written work, especially as the use of AI has become ubiquitous since chatbots have become freely available [1,2]. Consequently, critical concerns have arisen in the educational and academic sectors, where the reliability and authenticity of written work are fundamental.

According to a recent nationwide survey, nearly two-thirds of the German students reported using AI-based tools for their studies, with ChatGPT being the most commonly used chatbot [3]. ChatGPT, developed by OpenAI, an American AI research laboratory, is a state-of-the-art AI chatbot capable of assisting users with a wide range of tasks, from text generation to problem-solving [4,5]. Its capabilities have opened up significant opportunities for educational and academic contexts. For example, AI-based tools like ChatGPT can support tasks such as text analysis, translation, and proofreading for research purposes [6]. They also can provide support to students by enhancing their understanding of scientific methods, improving and refining written work, and assisting with examination preparation [3,7].

However, the widespread use of such tools raises concerns about their impact on students’ development of critical and independent thinking skills [8,9]. In addition, it is possible that ChatGPT could provide incomplete or inaccurate information, potentially leading to misunderstandings of academic concepts and topics [10,11]. Further concerns arise from the potential for academic dishonesty and plagiarism, particularly in the context of written assignments and academic essays [9,12].

The lack of clarity on how to handle such cases is putting universities in a quandary, leading to the first court cases and, more recently, to the University of Munich being vindicated in its decision to reject such written work [13]. In this case, an essay submitted as part of a Master’s application was rejected because it was “too well written,” raising suspicions that the text was likely generated by an AI tool such as ChatGPT [13]. This case highlights that AI-generated texts are often characterized by their seemingly perfected style of formulation that refers to the linguistic level [13,14], a characteristic that is known to be particularly pronounced and even more nuanced in English output texts compared with other languages such as German [15-18].

The relevance of the problem for medical studies is not obvious at first glance because medical students generally do not have to write long scientific texts on a regular basis during their studies, but usually only for their doctoral thesis. However, the increasing use of AI tools such as ChatGPT also poses challenges in the medical field, where assessments rely not only on linguistic quality but also on content accuracy. The potential misattribution of authorship in medical texts—such as research articles, patient information, or promotional materials—has particularly serious implications, as errors or inaccuracies in these contexts can have grave consequences. It can be assumed that medical texts do not fall as much within the scope of ChatGPT and are therefore more difficult to reproduce accurately, especially because AI authors have no “moral scruples” about concealing ignorance and replacing verified sources with falsified ones [19-22]. However, a few studies have addressed the problem of AI-generated content on medical texts [21-26], reporting that ChatGPT has, at times, managed to mislead medical professionals [26], which may suggest that familiarity with content plays a minor role in authorship identification.

Existing research on LLM-based text generators such as ChatGPT frequently focuses on their role in assisting the writing process rather than evaluating the quality and detectability of longer scientific texts [27-29]. In addition, studies often investigate the detection of texts written by chatbots using automatic tools or even detectors specifically designed for this purpose [18,25,27-30]. The detection rate of these detectors is often higher than that of human reviewers, but the accuracy can vary greatly depending on the text genre and the classifier used [14,31]. Moreover, linguistic features appear to be the most important subset of features influencing the performance of feature-based classifiers [2,5]. In the academic domain, educators still face the challenge of qualitatively assessing the authenticity of student texts, often without the aid of automated detection tools.

It is therefore of particular interest to determine how well human readers from the academic field can detect differences when directly comparing 2 texts on the same topic—an original student text and an AI-generated text—and which features stand out as particularly conspicuous and decisive for them. To better distinguish between the relevance of content-related and text-analytical attributes, we assembled a group of language experts from the humanities field alongside a group of medical experts specializing in pediatrics and neurology. Another key novelty and prerequisite of our study is the use of fully reproduced, longer scientific texts on medical topics written in German by medical students.

Therefore, we conducted a study to determine whether medical experts and humanities lecturers could distinguish between texts written by medical students and those generated by ChatGPT (specifically ChatGPT version 3.5, March 23). Unlike the interactive “Turing Test” [32], this task was not performed through a dialogue with a machine but rather through an internal, personal evaluation of 2 texts. We hypothesize that, in line with the Turing prophecy [32], the correct identification rate for AI-generated texts within a German medical sample is approximately 70%, with content familiarity playing a secondary role, while formal and linguistic features exert a greater influence.

Through a prospective analysis of longer German-language scientific texts written by students in the specialized health-related field of medicine, this study aims to provide new insights into AI-generated texts and the influence of content familiarity and linguistic expertise. The findings are intended to inform the development of guidelines to help lecturers (and others) recognize AI-generated texts, even in the absence of a comparable “original” text, and to contribute to future projects addressing the challenges posed by AI tools in academia.

## Methods

### Recruitment Process

This semirandomized controlled trial was conducted between May and August 2023 at the University Hospital of Pediatrics and Adolescent Medicine, St. Josef-Hospital, Ruhr University Bochum (RUB), Germany. To recruit participants, an open call was issued to the Department of Pediatrics and Adolescent Medicine and the Department of Neurology at the University Hospital Bergmannsheil (both RUB). Senior physicians and members of scientific working groups were invited to participate, ensuring the involvement of clinical experts familiar with the content of the texts. Participation was voluntary, with clinical employment or medical expertise serving as key inclusion criteria, along with an interest in scientific texts. In the next phase of recruitment, a call was made to the Faculty of Humanities at Ruhr University to include participants with experience in text reception and analysis, with teaching experience as an additional inclusion criterion.

### Design

Each participant received 2 pairs of texts, totaling 4 printed texts. Each pair consisted of 1 of 18 available term papers written by a medical student and a corresponding text generated by ChatGPT (version 3.5, March 2023), with the order of presentation randomized. The medical experts received 1 pair of texts on a topic closely related to their specialty and another pair on a less familiar topic. For example, a pediatrician received the text “Autoantibodies in Diabetes Mellitus,” while a neurologist received “Measurement of Aδ and C Fibers in Electrophysiology.” The second pair of texts covered a topic less directly related to their field of expertise.

Given the exploratory nature of the study, it became increasingly evident—only after completing the experimental phase with the medical experts—that the extent to which content familiarity influences the identification process needed further examination, particularly in comparison to formal and linguistic aspects.

As a result, a second phase of the study was initiated, involving a new group of experts with greater expertise in formal and linguistic analyses. This allowed for a comparison of results and evaluations across groups. In this group of humanities experts, each participant received the same 2 pairs of texts to ensure better comparability and verification of subject unfamiliarity. The first pair addressed a more general topic also familiar to nonmedical fields: “Iodine Deficiency.” The second pair analyzed a more specialized medical topic: “Autoantibodies in Diabetes Mellitus.”

Participants were asked to read a pair of texts and, based on their personal experience with student-written texts, decide within a week—without extensive research—which of the 2 they believed was generated by ChatGPT. To ensure the blinding of both interviewers and participants, the headers of the texts contained only a randomly generated 3-digit number and a “chatbot or student” checkbox. Before the subsequent interview, participants documented their decision by ticking the corresponding box for their chosen text version. They were also instructed not to discuss the task or the texts with one another.

About 1-2 weeks after the texts were distributed, participants were invited to a semistructured interview—conducted in person, by telephone, or via Zoom (Zoom Communications/Qumu Corporation)—to discuss their decisions, reasoning, and evaluations of the texts. Unblinding occurred after the interviews.

### Creation of the ChatGPT-Generated Versions

Eighteen German-language medical essays served as templates for the ChatGPT-generated texts. These essays were written by doctoral students from the University Pediatric Clinic and the Clinic for Neurology at Bergmannsheil University Hospital, RUB, Germany. They originated from the Doctoral Colloquium pool at the University Hospital of Pediatrics and Adolescent Medicine in Bochum. As part of the colloquium, each doctoral student is encouraged to write a scientific essay thematically related to their announced dissertation topic. To provide an initial experience with scientific research and writing—and to give the reviewing study coordinator a first impression of their academic level and skills—students do not receive specific instructions. For this study, all available German texts from this pool that were written before the general introduction of ChatGPT were considered, provided their authors consented to their use.

We used ChatGPT version 3.5, March 14 to replicate the texts. To generate a version with the same title and outline as the original papers while avoiding text breaks, 2 separate prompts were required to produce a continuous text from the introduction to the conclusion (Table 1).

For the main part, depending on the type of original paper, several commands were necessary. For example, see Table 2.

We then merged the individual sections to create a complete term paper, supplementing it with a bibliography that listed the sources provided by ChatGPT in sequential order. To ensure consistency, we harmonized the formatting of both ChatGPT-generated and student-written texts as much as possible, using the Arial font (size 11 for body text and size 12 for headings) with justified alignment. Sections or sentences specific to a student’s individual dissertation project were removed to maintain general applicability. However, we did not alter the choice of words, sentence structure, punctuation, spelling, or citation style.

**Table 1:** Prompts used to create ChatGPT text.

German (original prompt)	English (translation)
“Schreibe bitte einen Abschnitt über das Thema [TITEL DES TEILTHEMAS] der [wissenschaftlichen/medizinischen] Hausarbeit [TITEL DER HAUSARBEIT].” “Belege Deine Aussagen mit Quellen, die bei Pubmed auffindbar sind.”	“Please write a section on the topic [NAME OF SUBTOPIC] of the [scientific/medical] term paper [NAME OF TERM PAPER].” “Support your statements with sources that can be found on PubMed.”

**Table 2:** Additional instructions used to create ChatGPT text.

German (original prompt)	English (translation)
“Schreibe einen Abschnitt zum Thema ‘Potenziell reversible Pathomechanismen als mögliche Ursachen von Hyposmie oder Anosmie bei Kindern’ der wissenschaftlichen Hausarbeit ‘Ursachen und Diagnostik von Riechstörungen bei Kindern und Jugendlichen’, der an den vorherigen Abschnitt anknüpft.”	“Write a section on the topic ‘Potentially reversible pathomechanism as possible causes of hyposmia or anosmia in children’ of the scientific paper ‘Causes and diagnostics of olfactory dysfunction in children and adolescents’, which ties in with the previous section.”

### Data Assessment During the Interview

The medical expert group was interviewed by 2 blinded interviewers (BD and JSB), while the humanities expert group was interviewed by a partially blinded interviewer (CM). Initially, participants provided demographic information, including age, experience in academic and student teaching, academic qualifications, publication history, and prior experience with ChatGPT. They were then asked to assess how well the following questions were addressed in each text, using the German grading system from 1 (very good) to 6 (unsatisfactory) (see Multimedia Appendix 1 for details). How would you rate (1) linguistic fluency, (2) scientific quality (eg, are the re-definitions scientifically derived and are studies cited that lead to certain conclusions?), (3) internal logic, (4) description of the limitations of current knowledge, (5) future research questions, and (6) citations and references of the text? Participants were then asked to identify which text version, using the corresponding 3-digit number, they had categorized as being generated by ChatGPT and to list the key reasons for their decision, which the interviewer recorded using keywords. Next, they rated their confidence in their decision on a scale from 1 (very confident) to 6 (not confident at all). After this initial assessment, participants were unblinded and informed which text had been written by a student and which by ChatGPT. In cases of misidentification, they were asked about their suspected reasons, which the interviewer also documented using keywords.

### Construction of Categories

Beyond the identification rate and the text evaluations by each group, a qualitative analysis of participants’ statements regarding their reasoning for assigning authorship proved essential. This deeper analysis aimed to examine the influence of content-related versus formal-linguistic aspects and to better attribute global features to either student or chatbot authorship. For this purpose, the free-text responses (recorded by interviewers using keywords) were first thematically clustered based on the terms mentioned (see sample statements in the *Free-Text Analysis* section). Subsequently, through multiturn discussions between medical and linguistic experts, these thematic clusters were refined into distinct, nonoverlapping categories that encompassed all interviewee statements while reducing redundancy and multiple classifications.

Many of these categories align with standard text-analytical frameworks, which typically cover a broad range of attributes, including morphology, syntax, style, structure, coherence and cohesion, content quality, form, and even sociolinguistic aspects [33-35]. However, the categories derived in this study are directly based on the text types used in the experiments. As a result, they provide a more precise representation of the emerging and still undefined text type “AI-generated” and are therefore preferable (see Multimedia Appendix 2 for an overview of the categories).

### Statistical Analysis

Data were analyzed using Microsoft Excel, SPSS version 29.3 (IBM Corp.), and R-4.1.2 (R Foundation). Descriptive statistics are presented as numbers (n) and percentages or as means (SD), where appropriate. Univariate odds ratios and 95% CIs from the Fisher exact test were used to examine the association between demographic markers, participants’ field (medicine or humanities), and their expertise with the likelihood of correctly identifying a text’s source. The relationship between interview scores and response accuracy was assessed using the 2-sided Wilcoxon signed rank test for paired values. Additionally, correlations among all 5 responses were tested using a Friedman 2-way analysis of variance for ranks with Bonferroni correction.

To analyze how participants attempted to identify the machine-generated text, we modeled the association of the derived categories (items) from the interviews based on their likelihood of being mentioned in the context of a chatbot-generated text. For each participant and interview, we recorded whether an item was cited in reference to a perceived chatbot text or a perceived student text. This association was analyzed using repeated-measures logistic regression, incorporating a random participant and sequence effect. The model was further adjusted for age group, the expert group (medical vs humanities), and prior experience with ChatGPT (binary).

### Ethical Considerations

An application for the study project was submitted to the Ethics Committee of the Medical Faculty at RUB (reference number 23-7837; April 2023). As the study did not involve direct research on human participants or patient data, the committee informed us that ethical approval was not required.

## Results

### Interviewee Sample

The biographical data of the 22 participating physicians (14 pediatricians, 3 nutritionists, 4 neurologists, and 1 neuroscientist) and 13 humanities scholars (8 literary scholars, 3 Germanists or linguists, 1 classical philologist, and 1 Romance philologist) are presented in Table 3.

As there were more participating experts than available term papers, 3 pairs of texts were each assessed by 3 or 4 medical experts.

At the time of the survey, only one-fifth of the participants reported having prior experience with ChatGPT. As the number of participating experts exceeded the number of available term papers, 3 pairs of texts were each assessed by 3 or 4 medical experts.

**Table 3:** Interviewee sample.

Characteristics		All participants (N=35)	Medical experts (n=22)	Humanities experts (n=13)
**Age (years), n (%)**				
	<40	17 (49)	13 (59)	4 (31)
	≥40	18 (51)	9 (41)	9 (69)
**Experience in academic teaching^a^ (years), n (%)**				
	None	2 (6)	2 (9)	N/A^b^
	<5	8 (23)	8 (36)	N/A
	≥5	25 (71)	12 (55)	13 (100)
**PhD/professorship, n (%)**				
	Yes	28 (80)	18 (82)	10 (77)
**Authorship in a publication, n (%)**				
	Yes	32 (91)	20 (91)	12 (92)
**Experience with ChatGPT^a^, n (%)**				
	Yes	7 (20)	2 (9)	5 (38)
	Only a little	7 (20)	4 (18)	3 (23)
	No	21 (60)	16 (73)	5 (38)

^a^Self-assessed.

^b^N/A: not applicable.

### Detection Rate

With 35 participants evaluating 2 text pairs each—excluding 1 misaligned and omitted case—a total of 69 decision rounds were conducted. In 48 out of 69 (70%) decision rounds, participants correctly identified the authorship of the texts. Medical and humanities experts showed a slight but nonsignificant difference in detection rates, with medical experts correctly identifying 31 out of 43 (72%) decision rounds compared with 17 out of 26 (65%) decision rounds by humanities experts (odds ratio 1.37, 95% CI 0.5-3.9). Among the 35 participants, 21 (60%) misidentified the authorship of at least one text pair, including 12 medical experts. Additionally, 5 (14%) participants, including 3 physicians, misidentified both text pairs.

Notably, familiarity with the topic did not significantly impact identification accuracy (Table 4), nor did personal characteristics such as age, academic qualifications, or years of teaching experience. However, younger participants without advanced academic titles showed a slight tendency to better identify ChatGPT-generated texts. Confidence in participants’ own judgments did not differ significantly across groups (odds ratio 0.6, 95% CI 0.2-1.79).

**Table 4:** Characteristics of the participants with correct and incorrect decisions about the authorship of the respective text.

Characteristics			All participants							Medical experts							Humanities experts						
			Decision							Decision							Decision						
			Correct		False		OR^a^ (95% CI)		Correct			False		OR (95% CI)		Correct			False		OR (95% CI)		
Tests, n (%)			48 (70)		21 (30)		N/A^b^		31 (72)			12 (28)		N/A		17 (65)			9 (35)				
**Age (years), n (%)**																							
	<40	24 (73)		9 (27)		N/A		17 (68)			8 (32)		N/A		7 (88)			1 (12)		N/A			
	≥40	24 (67)		12 (33)		0.75 (0.27-2.11)		14 (78)			4 (22)		1.65 (0.41-6.63)		10 (56)			8 (44)		0.18 (0.02-1.77)			
**PhD/professorship, n (%)**																							
	Yes	37 (67)		18 (33)		N/A		25 (71)			10 (29)		N/A		12 (60)			8 (40)		N/A			
	No	11 (78.6)		3 (21.4)		1.78 (0.44-7.2)		6 (75)			2 (25)		1.2 (0.21-6.98)		5 (83.3)			1 (16.7)		3.33 (0.33-34.12)			
**Experience in academic teaching^c^ (years), n (%)**																							
	≥5	30 (67)		15 (33)		N/A		16 (70)			7 (30)		N/A		14 (64)			8 (36)		N/A			
	<5 or none	18 (75)		6 (25)		1.5 (0.49-4.56)		15 (75)			5 (25)		1.31 (0.34-5.05)		3 (75)			1 (25)		1.71 (0.15-19.36)			
**Authorship in a publication, n (%)**																							
	Yes	44 (70)		19 (30)		N/A		28 (72)			11 (28)		N/A		16 (67)			8 (33)		N/A			
	No	4 (67)		2 (33)		0.86 (0.15-5.12)		3 (75)			1 (25)		1.18 (0.11-12.59)		1 (50)			1 (50)		0.5 (0.03-9.08)			
**Experience with ChatGPT^c^, n (%)**																							
	Yes	20 (74)		7 (26)		N/A		9 (82)			2 (18)		N/A		11 (69)			5 (31)		N/A			
	No	28 (67)		14 (33)		0.7 (0.24-2.05)		22 (69)			10 (31)		0.49 (0.09-2.69)		6 (60)			4 (40)		0.68 (0.13-3.55)			
**Text pair (sequence), n (%)**																							
	First	23 (66)		12 (34)		N/A		16 (73)			6 (27)		N/A		7 (54)			6 (46)		N/A			
	Second	25 (74)		9 (26)		0.75 (0.27-2.09)		15 (71)			6 (29)		0.94 (0.25-3.56)		10 (77)			3 (23)		2.86 (0.53-15.47)			
**Familiar with the topic, n (%)**																							
	More	25 (68)		12 (32)		N/A		18 (75)			6 (25)		N/A		7 (54)			6 (46)		N/A			
	Less	23 (72)		9 (28)		1.01 (0.36-2.8)		13 (68)			6 (32)		0.72 (0.19-2.75)		10 (77)			3 (23)		2.86 (0.53-15.47)			
**Self-confidence in the decision^c^, n (%)**																							
	Rather sure	35 (73)		13 (27)		N/A		22 (76)			7 (24)		N/A		13 (68)			6 (32)		N/A			
	Unsure	13 (62)		8 (38)		0.6 (0.2-1.79)		9 (64)			5 (36)		0.57 (0.14-2.29)		4 (57)			3 (43)		0.62 (0.1-3.66)			

^a^OR: odds ratio for the correct decision.

^b^N/A: not applicable.

^c^Self-assessed.

### Interview Analysis

**Figure 1:**
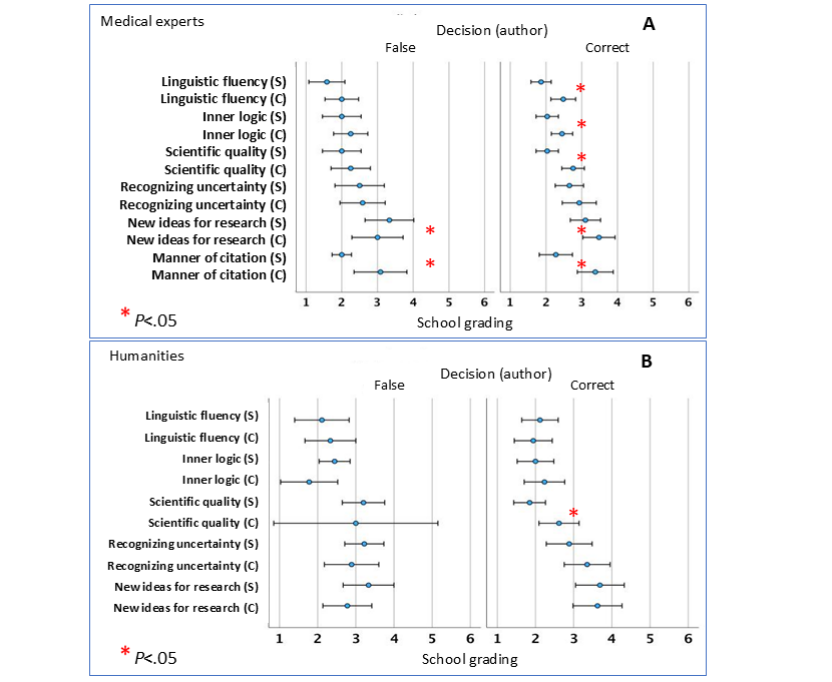
Association of mean school grade (using German school grades 1=very good to 6=unsatisfactory) and correctness of authoring identification in (A) medical experts and (B) humanities experts. Participants were not yet unblinded at the time of assessment (see Multimedia Appendix 1 for details). Left side: incorrect attribution, right side: correct attribution of authorship. *P<.05 (2-sided Wilcoxon signed rank test). C: chatbot-generated text; S: student text.

When authorship was correctly identified, texts written by medical students received notably higher ratings for stylistic fluency, the internal logic of argumentation, and scientific quality (see Figure 1A, right-hand side). These differences were less pronounced in the assessment of knowledge limitations and future research directions. Regardless of correct identification, the way sources were cited was consistently rated higher in student-authored texts (Figure 1). Among humanities experts, score differences between correctly and incorrectly classified texts were minimal, though the academic quality of student-written texts was still rated significantly (*r*=0.336, *P*=.009) higher overall (Figure 1B, right-hand side). Many participants who misidentified the authorship attributed their errors to either underestimating the quality of student work or overestimating ChatGPT’s capabilities—particularly in terms of logical coherence, the presentation of scientific knowledge limitations, and the formulation of new research ideas.

### Free-Text Analysis

We categorized the 187 freely formulated reasons participants provided for their decisions into 1 of 12 derived categories (see Multimedia Appendix 2). Three categories were excluded from statistical analysis due to their low frequency: inconsistency of writing style (n=4), other issues (n=4), and errors in content (n=3). Notably, all 3 content-error attributions came from the medical expert group. Of the remaining 176 statements, 88 (50%) were contributed by medical experts and 88 (50%) by humanities scholars (Table 5).

The experiment revealed that significantly more statements were made about (suspected) ChatGPT-generated texts (130/176, 73.9%) than about student-written texts, regardless of whether the suspicion was correct (Table 5). Sample statements from both groups are provided in Tables 6 and 7. Medical experts’ explanations were often concise, frequently critiquing a “superficial” style with “unnecessary additional information.” By contrast, humanities experts tended to provide more detailed justifications, describing characteristics such as “smooth style” and “redundancies.”

**Figure 2:**
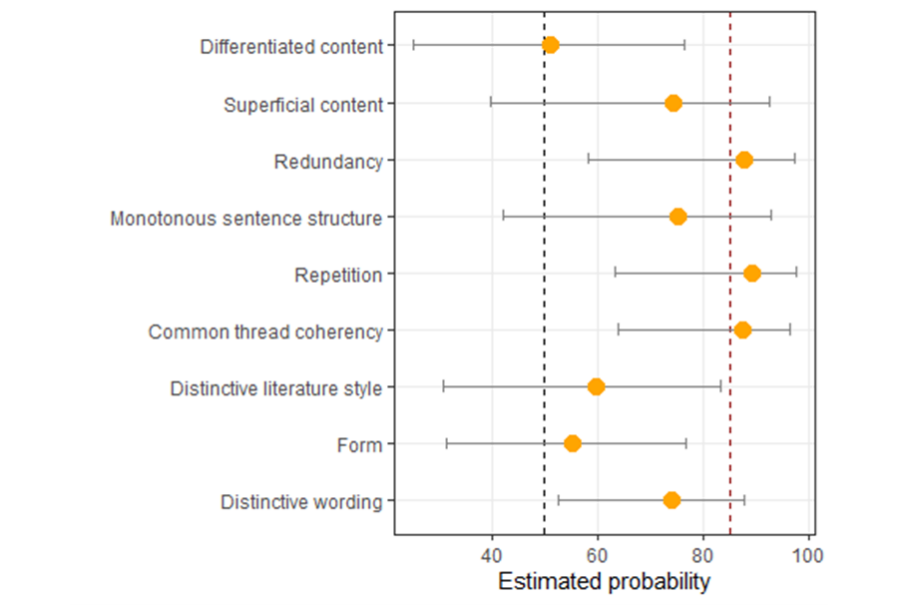
Estimated probability of thinking of ChatGPT as authorship.

We analyzed the likelihood of specific categories being mentioned in reference to texts suspected to be generated by ChatGPT. The results indicate that “redundancy” (12/14, 86%, associated with GPT vs 2/14, 14%, with student texts), “repetition” (20/22, 91% vs 2/22, 9%), and “common thread and coherence” (21/24, 88% vs 3/24, 13%) were the most frequently cited characteristics (Figure 2).

**Table 5:** The remaining 9 categories and item frequency by presumed nature of the text (for a detailed explanation in German and English, see Multimedia Appendix 2).

Item	Category	How often mentioned				
		Overall, n (%)	Humanities		Medical experts	
			Chatbot, n (%)	Students, n (%)	Chatbot, n (%)	Students, n (%)
		176 (100)	59 (33.5)	29 (16.5)	71 (40.3)	17 (9.7)
1	Differentiated content	16 (9.1)	4 (6.8)	4 (13.8)	4 (5.6)	4 (23.5)
2	Superficial content	13 (7.4)	2 (3.4)	2 (6.9)	8 (11.3)	1 (5.9)
3	Redundancy	14 (8.0)	7 (11.9)	2 (6.9)	5 (7.0)	N/A^a^
4	Monotonous structure of sentences	14 (8.0)	6 (10.2)	4 (13.8)	4 (5.6)	N/A
5	Repetition	22 (12.5)	8 (13.6)	1 (3.4)	12 (16.9)	1 (5.9)
6	Common thread coherency	24 (13.6)	10 (16.9)	2 (6.9)	11 (15.5)	1 (5.9)
7	Distinctive literature style	16 (9.1)	3 (5.1)	2 (6.9)	8 (11.3)	3 (17.6)
8	Form	25 (14.2)	6 (10.2)	6 (20.7)	9 (12.7)	4 (23.5)
9	Distinctive Wording	32 (18.2)	13 (22.0)	6 (20.7)	10 (14.1)	3 (17.6)

^a^N/A: not applicable.

**Table 6:** Excerpt from the statements of the medical expert group on the main reasons for choosing ChatGPT as the author.

German (original statement)	English (translation)
“‚erschien zu perfekt geschrieben, oberflächlich‘”	“‚seemed too perfectly written, superficial’”
“‚unnützes Wissen, Zusatzinfos, die nicht notwendig für die Arbeit wären‘”	“‚useless knowledge, additional information that is not necessary for the work‘”
“‚fehlender roter Faden, fehlende Kontinuität der Logik’”	“‚lack of common thread, lack of continuity of logic’”

**Table 7:** Excerpt from the statements of the humanities expert group on the main reasons for choosing ChatGPT as the author.

German (original statement)	English (translation)
“‚fehlende Kohärenz, Redundanz, Monotonie […] ist sehr redundant, wiederholt Formulierungen teils mehrfach in Variationen. Der Definitionsteil wirkt reihend, stilistisch homogen, […] teils erfährt man, was man sich hätte denken können, […]. Der letzte Absatz wiederholt in etwa, was vorher da stand - was so wirkt, als hätte er diesen schon 'vergessen'.”	“‚lack of coherence, redundancy, monotony [...] is very redundant, repeats formulations sometimes several times in variations. The definition section appears to be sequential, stylistically homogeneous, [...] partly one learns what one could have imagined, [...]. The last paragraph roughly repeats what was there before - which makes it seem as if he had already 'forgotten' it.”
“‚Smooth, fließende Übergänge, aber übertextlich schlechter, d.h. gesamt Kohärenz schlechter (Wiederholung), habe nichts gelernt‘.”	“‚Smooth, fluent transitions, but overtextually worse, i.e. overall coherence worse (repetition), 'haven't learned anything'.”
“‚Wiederholung vieler Sätze und Inhalte; ausgeprägte Tendenz zu bestimmten schablonenartigen Formulierungen im Sinne einer ‘Anmoderation’, z.T. phrasenhaft ohne Inhalt. Optisch gute Gliederung (vorgegeben durch die Überschriften), jedoch roter Faden nicht gut erkennbar, vieles wirkt lediglich wie aufgelistete Einzelformationen. […] Nennung vieler Quellen, deren Zuordnung zu einzelnen Aussagen ist oft nicht konkret.”	“‚Repetition of many sentences and contents; pronounced tendency towards certain template-like formulations in the sense of a ‘presentation’, sometimes phrase-like without content. Visually well structured (given by the headings), but a common thread is not easily recognizable, many things appear to be merely a list of individual formations. [...] Mention of many sources, their assignment to individual statements is often not concrete.”

## Discussion

### Overview

This analysis offers insights into the current identification rate of AI-generated texts and their evaluation compared with medical student texts by 2 different expert groups. It also provides an initial overview of the decision-making processes of medical and humanities experts during these assessments. Our findings suggest that both medical and humanities experts can effectively identify ChatGPT-generated texts in medical contexts and that linguistic and stylistic features play a significant role in distinguishing AI-generated from human-written texts, regardless of content familiarity. This supports the broader notion that linguistic analysis is crucial in identifying AI-generated text, aligning with foundational theories in human-robot interaction, such as Turing’s predictions [32].

### Identification Rate

In the 1950s, Alan Turing [32] predicted that within 50 years, AI would advance to the point where the likelihood of identifying a machine as nonhuman in a dialogue or an “imitation game” would be no more than 70% [32]. With a slight delay of about 20 years, his prediction was almost precisely fulfilled in an online game inspired by the Turing Test [5]. Unlike Turing’s method and the large-scale Israeli study, our research did not involve direct dialogue between humans and machines [5,32]. However, when participants were presented with 2 texts of different authorship, an internal dialogue was essential for making an authorship determination. Ultimately, our study’s main finding aligns almost exactly with Turing’s prediction: only in 48 out of 69 (70%) decision rounds, participants correctly identified the ChatGPT-generated text. This accuracy rate remained consistent regardless of whether participants were experts in the content of the text or in linguistic analysis, and irrespective of their prior experience with ChatGPT. Notably, familiarity with the subject matter did not appear to be a decisive factor, as humanities experts performed similarly to medical experts who specialized in the respective topics. Moreover, at the individual participant level, no significant differences were found between the 2 expert groups in terms of their proximity to the text’s subject matter.

A Chinese study by Ma et al [2], which also examined the identification rate of chatbot-generated texts, reported similar findings, with approximately 66% of texts correctly identified. This study analyzed around 40 scientific texts, including 20 scientific paper abstracts and 20 wiki item descriptions, assessed by 2 PhD students with a background in computer science [2]. Ma et al [2] also highlighted notable differences in writing style between AI-generated and human-written scientific texts, a conclusion that aligns with our findings. In our study, participants primarily based their decisions on text-analytical features, while content errors influenced their judgment in only 3 instances.

The study by Waltzer et al [36], which closely resembles our research in design, reported similar findings. In their study, 140 college instructors were presented with pairs of essays and correctly identified the ChatGPT-generated text 70% of the time. Like our results, Waltzer et al [36] found that neither prior experience with ChatGPT nor subject-specific expertise—measured by self-reported familiarity with the topic—significantly improved identification accuracy [36]. However, a key difference is that their study analyzed English-language essays written for a psychology program, whereas our research focused on German-language texts authored by medical students [36].

### Performance

The evaluation of a text can focus on different levels and aspects, often emphasizing either content or linguistic features. Currently, AI programs such as ChatGPT are recognized for their seemingly perfected linguistic style [13,14]. A notable case at a German university (TU Munich) illustrates this: an essay submitted as part of a Master’s application was rejected—and this decision was upheld by a court—on the grounds that it was “too well written,” strongly suggesting AI authorship [13]. However, it is important to note that this essay was written in English [13]. While ChatGPT is also proficient in translating languages such as German and Chinese [15], its performance in German differs from English. Research suggests that AI-generated texts tend to be more nuanced and varied in English than in German [16,17]. This discrepancy is likely due to the greater availability of digital data in English, which results in more refined and contextually accurate outputs. Nevertheless, AI language models continuously improve as they interact with users, enhancing their capabilities in non–English languages over time.

Interestingly, the humanities group, despite their focus on linguistic features, identified ChatGPT-generated texts less accurately than the medical expert group—though this difference was not statistically significant. Notably, humanities experts rated the linguistic quality of ChatGPT texts higher than those written by medical students, a contrast that was significant compared with the evaluations of the medical experts. The decision-making process behind text identification revealed key patterns: participants were more likely to suspect a human author when encountering spelling and grammatical errors, greater variation in sentence structure, medical-specific terminology, a writing style aimed at a professional readership, or shifts in citation style.

An “AI author,” by contrast, was suspected if there was a monotonous sentence structure, partly “English” grammar, a “smooth” wording style, that is, good readability/understandability, but overall more superficial, an intended less professional readership, better overall formal structure of the text (derivation, outline, weft), frequent repetitions, and a lack of supra-textual coherence of the argumentation in contrast to the coherent and easily comprehensible sequence of arguments within individual paragraphs.

Many studies explored the identifiability of chatbot-generated text using machine learning–based detectors, a subset of AI technologies [27-29]. These detectors often achieve higher identification rates than human evaluators. However, direct human comparison is rarely included, and accuracy and *F*_1_-scores vary significantly depending on the text genre and the specific machine learning classifier used. For instance, when various LLM-based classifiers are applied to different data sets, their accuracy ranges from 70% (DetectGPT classifier on Wikipedia articles) to 97% (GPT-Pat classifier on COVID-19–related question-answer data sets). Similarly, perplexity-based classifiers achieve around 70% accuracy on ACL paper abstracts, whereas RoBERTa (Robustly Optimized BERT Pretraining Approach)–based classifiers reach up to 97% on COVID-19–related data sets [37].

Another challenge is that while these tools are generally reliable in detecting AI-generated text, they are not always sufficiently accurate in identifying human-authored text. This suggests that the tools may struggle with the complexity of human writing while also highlighting a key limitation—especially in cases where a lecturer, for example, must evaluate a single piece of writing without comparison [14,31].

Our study also compares the performance of medical students and ChatGPT. Notably, the texts written by medical students received higher professional evaluations. However, the humanities experts specifically rated the linguistic quality of ChatGPT-generated texts more favorably. Additionally, when comparing ChatGPT’s performance with that of medical students in an examination setting—such as in the study by Huh [38]—ChatGPT performed worse than medical students [31]. In a parasitology examination, ChatGPT correctly answered 60.8% of the questions, whereas the average score among 77 medical students was significantly higher at 90.8% [38]. In comparison, a German study by Friederichs et al [39] found that ChatGPT correctly answered two-thirds of all multiple-choice questions at the level of the German state licensing examination in the Progress Test Medicine. It even outperformed most medical students in their first 3 years and performed comparably to students in the later stages of their studies [39]. Our study also revealed that participants who overestimated ChatGPT’s writing capabilities and underestimated those of the students were more likely to misidentify the author. This misconception was particularly evident in cases where participants misclassified texts in both sessions, suggesting that their biased perception significantly influenced their decisions.

### Interpretation

Our study demonstrates that the identification rate predicted by Turing holds within a group primarily engaged in student teaching and academic writing. Our findings confirm the expectation that linguistic features play a more significant role in identifying AI-generated texts than content familiarity or specialized expertise. In both expert groups, text-analytical features were the primary factors influencing their decisions. This aligns with the emerging field of stylometric analysis, which is increasingly being applied to the detection of AI-generated content [40]. ChatGPT-generated text, especially in comparison to authors from the (fictional) literature domain, exhibits limited stylistic variety [41]. Notably, there was no significant difference in the identification rate between the 2 expert groups, despite 1 being more familiar with the subject matter. Higher proximity to the topic was also not a predictive factor at the individual participant level. Instead, certain linguistic characteristics played a key role in the decision-making process and were consistently associated with AI-generated texts. In particular, redundancy, repetition, and a lack of coherence were distinctive features attributed to ChatGPT-generated texts. While these traits influenced the perception of AI authorship, they ultimately did not prove to be reliable predictors for correct identification. The linguistic features of ChatGPT-generated texts are often perceived as superior due to their smoother wording and better structural organization. This aligns with findings from [42], which indicate that AI-generated texts tend to exhibit relatively low lexical density, high reading ease, and frequent use of the simple present tense. Whether these linguistic characteristics, if systematically outlined in a manual and provided to participants beforehand, could enhance identification accuracy remains an open question. However, this presents an intriguing avenue for future research.

### Limitations

While numerous studies are currently investigating the performance of LLM-based text generators such as ChatGPT, many focus primarily on their assistive role in the writing process rather than assessing the quality of fully generated long-form scientific texts. A key contribution of our study is that it examines complete, AI-generated scientific texts rather than partial outputs. Additionally, instead of relying on specialized AI detection tools, we analyze how individuals working in academia recognize such texts without assistance and how they evaluate their performance while identifying distinct linguistic features. This study enables the compilation of categorical features that could aid in identifying AI-generated text in both academic and everyday reading. However, limitations in generalizability arise due to the relatively small sample size and the exclusive use of a single AI model, ChatGPT version 3.5. Nevertheless, for an exploratory study, we do not consider this a critical issue. Additionally, participants were aware that 1 of the texts had to be AI-generated, raising the question of whether they would have identified an AI-authored text without this prior knowledge. A further limitation arises from the use of different interviewers for the 2 expert groups, who also differed methodologically in terms of blinding. However, it should be noted that the decision to identify the authors was always made before the interview process. Additionally, the interview was transcribed in bullet points, so some information may have been lost in this process. Finally, the dynamic nature of development should also be acknowledged, as ChatGPT, like other AI programs, is continuously being developed and improved.

### Conclusion

Our study shows that linguistic and text-analytical features, in particular, play a role in the decision-making process for correctly identifying a chatbot author. In our sample, both nonspecialists and specialists identified AI-generated texts with an accuracy rate of approximately 70% (48/69). Further quasi-experimental studies using texts from other academic disciplines should be conducted to determine whether instructions based on these features can enhance lecturers’ ability to distinguish between student-authored and AI-generated work.

A follow-up study could be conducted in a few years to track the evolution of AI-generated text identification and examine whether identification success changes as AI technology and tools advance.

## Appendix

Multimedia Appendix 1 Questionnaire with English translation and abbreviation label.

Multimedia Appendix 2 Categories for the qualitative analysis, created from the various reasons given in free form for the decision on the authorship of a text.

## Abbreviations

AI: artificial intelligence
LLM: large language model
RoBERTa: Robustly Optimized BERT Pretraining Approach
RUB: Ruhr University Bochum
