# Supplementary material for: Detecting Artificial Intelligence–Generated Versus Human-Written Medical Student Essays: Semirandomized Controlled Study
Source: JMIR Med Educ. 2025 Mar 3;11:e62779. doi: 10.2196/62779 (PMC11914838; doi:10.2196/62779)
Supplement: Multimedia Appendix 1 [file mededu_v11i1e62779_app1.docx]

**Multimedia Appendix 1**

Table 1: Questionnaire with English translation and abbreviation label.

| **German question** | **English translation** | **Abbreviation** |
| --- | --- | --- |
| Wie bewerten Sie die sprachliche Qualität des Textes? | How would you judge the linguistic quality of the work | Linguistic fluency |
| Wie bewerten Sie die wissenschaftliche Qualität? z. b. werden die Definitionen wissenschaftlich hergeleitet und Untersuchungen genannt, die zu bestimmten Schlussfolgerungen führen? | How would you judge the scientific quality (e.g. the derivation of definitions, justification of conclusions from previous studies | Scientific quality |
| Wie bewerten Sie die Argumentationsfolge und innere Logik des Textes | How would you judge the internal logic of the text and order of argumentation | Inner logic |
| Wie bewerten Sie die Darstellung der Grenzen des bisherigen Wissens? | How would you judge the presentation of the limits of knowledge, i.e., the state of non-knowledge, | Recognizing uncertainty |
| Wie bewerten Sie, inwieweit Ideen für zukünftige Forschung aufgeworfen werden? | How would you judge the mention of ideas for future research, | New ideas for research |
| Wie bewerten Sie die Zitierweise und Qualität der Quellen? | How would you judge the style or manner of citations | Citation und references |
