# Supplementary material for: Detecting Artificial Intelligence–Generated Versus Human-Written Medical Student Essays: Semirandomized Controlled Study
Source: JMIR Med Educ. 2025 Mar 3;11:e62779. doi: 10.2196/62779 (PMC11914838; doi:10.2196/62779)
Supplement: Multimedia Appendix 2 [file mededu_v11i1e62779_app2.docx]

**Multimedia Appendix 2**

Table 2: Categories for the qualitative analysis, created from the various reasons given in free form for the decision on the authorship of a text.

| **Item** | **label** | **N (%)** | **English Wording** | **German Wording** |
| --- | --- | --- | --- | --- |
| 1 | **Differentiated content** | 16 (9.1%) | (Topic)-specific argumentation; specific content; differentiation; information content high; depth of information (specific) high; information quantity large, information on the implementation of the studies; detail of the explanations; reference to work processes, embedding in the research/historical context; detailed descriptions | (Themen)-spezifische Argumentation; Spez. Inhalte; Differenziertheit; Informationsgehalt hoch; Informationstiefe (spezifisch) hoch; Informationsmenge groß, Informationen zur Durchführung der Studien; Detailliertheit der Ausführungen; Bezug zu Arbeitsabläufen, Einbettung in den Forschung-/ historischen Kontext; detailreiche Beschreibungen |
| 2 | **Superficial content** | 13 (7.4%) | Argumentation rather general; Undifferentiation; information content low; no information on the implementation of the studies; depth of information superficial; embedding in the research/historical context superficial, e.g. negations of statements on 1 | Argumentation eher allgemein; Undifferenziertheit; Informationsgehalt niedrig; keine Information zur Durchführung der Studien; Informationstiefe oberflächlich; Einbettung in den Forschung-/ historischen Kontext oberflächlich; u.a. Verneinungen von Aussagen zu 1 |
| 3 | **Redundancy** | 14 (8.0%) | useless information on content; content-related redundancies (at sentence level); linguistic redundancy (at word/phrase level); filler sentences/phrase language | unnütze Informationen zum Inhalt; inhaltliche Redundanzen (auf Satzebene); sprachliche Redundanz (auf Wort-/Phrasenebene); Füllsätze/ Phrasen- Sprache |
| 4 | **Monotonous**  **sentence structure** | 14 (8.0%) | sentence structure uniform or varied; paragraph structure; rhetorical clichés | Satzstruktur einheitlich oder variantenreich; Absatzstruktur; Rhetorische Floskeln |
| 5 | **Repetition** | 22 (12.5%) | Repetition of sentence elements (phrases) or words | Wiederholungen von Satzelemente (Phrasen) oder Wörter |
| 6 | **Common thread coherency** | 24 (13.6%) | Connection of text modules (= common thread); textual inconsistency (= copied together); logic of statements; conclusion (thesis-like vs. enumerative); text structure; clear structuring elements vs. jumpy/random; internal structuring of paragraphs (variations); linking of paragraphs vs. independence of statements | Verbindung der Textbausteine (= roter Faden); Textuelle Inkonsistenz (= zusammenkopiert); Logik der Ausführungen; Schlussfolgerung (thesenhaft vs. aufzählend); Textstruktur; klare Gliederungselemente vs. sprunghaft/ zufällig; Binnengliederung der Absätze (Variationen); Verknüpfung der Absätze vs. Unabhängigkeit der Aussagen |
| 7 | **Distinctive literature style** | 16 (9.1%) | journal titles (translated?); Researchers working at RUB are cited; number of sources; specificity of sources; Textbooks/ student teaching texts ("Duale Reihe"); German and English language sources; guidelines; invented sources; timeliness of the sources; traceability of the attribution of sources | Zeitschriftentitel (übersetzt?)  An der RUB tätige Forscher werden zitiert; Anzahl der Quellen; Spezifität der Quellen; Lehrbücher/ Studentische Lehrtexte („Duale Reihe“); deutsch- und englischsprachige Quellen; Leitlinien;  erfundene Quellen; Aktualität der Quellen; Nachvollziehbarkeit der Zuordnung der Quellen |
| 8 | **Form** | 25 (14.2%) | Quality of citations; inconsistency of citation style (uniformity/quality); errors in the list of sources (omissions, repetitions, uniformity); incorrect spelling/grammar; formatting changes | Qualität der Zitate; Inkonsistenz der Zitierweise (Einheitlichkeit/ Qualität); Fehler im Quellenverzeichnis (Auslassungen, Wiederholungen, Einheitlichkeit); Fehlerhafte Rechtschreibung/ Grammatik; Formatierungswechsel |
| 9 | **Distinctive**  **wording** | 32 (18.2%) | Vocabulary (correctness/ variation/ conciseness); unusual plural forms; Syntax: e.g. incomplete sentences; Smooth wording; Target group-specific wording | Wortschatz (Korrektheit/ Variation/ Prägnanz); ungewöhnliche Pluralformen; Syntax: z.B. unvollständige Sätze; Flüssigere Formulierungen („Smooth Wording“); Zielgruppen-spezifisches Wording |
| 10^a^ | **Inconsistency of authoring style** | 4  (2.3% | Abbreviations (consistency/ introduction/ repetition); Language changes (for technical terms); Variations in the spelling of technical terms; Tense (consistency/ correctness) | Abkürzungen (Einheitlichkeit/ Einführung/ Wiederholungen); Sprachwechsel (bei Fachbegriffen); Varianten in der Schreibweise von Fachbegriffen; Tempus (Einheitlichkeit/ Korrektheit) |
| 11^a^ | **Incorrect content** | 3  (1.7%) | Content errors | Inhaltliche Fehler |
| 12^a^ | **Other**  **reasons** | 4  (2.3% | Illustrations; Self-confidence of the writer; Blanks | Abbildungen; Selbstsicherheit des Schreibenden; Leerzeichen |

^a^ No. 10-12 were excluded due to less than 5 notations.
